# Incidence and prognostic implications of acute kidney injury based on the RIFLE criteria at the time of admission to an Indian ICU

**DOI:** 10.1186/cc11705

**Published:** 2012-11-14

**Authors:** D Juneja, P Nasa, O Singh, Y Javeri, S Garg

**Affiliations:** 1 Max Super Speciality Hospital Saket, New Delhi, India

## Background

Acute kidney injury (AKI) is an important predictor of outcome in patients admitted to ICUs. The Acute Dialysis Quality Initiative (ADQI) Group has defined and stratified AKI according to the Risk, Injury, Failure, Loss and End-stage renal disease (RIFLE) criteria [1]. We aimed to assess the ability of the RIFLE criteria to predict mortality in critically ill patients admitted to a medical ICU.

## Methods

A retrospective cohort study in an eight-bed medical ICU of a tertiary care hospital over a period of 16 months. Data regarding patient demographics and ICU course including need for organ support and length of stay were recorded. We classified each patient according to their RIFLE class using admission creatinine values (no AKI: <1.5 × baseline, Risk: ≥1.5 × baseline, Injury: ≥2 × baseline or Failure: ≥3 × baseline), as previously described [1]. Qualitative data were analyzed using the chi-squared or Fisher exact test as appropriate and quantitative data were analyzed using Student's *t *test. Inter-group and intra-group comparison for quantitative data was done by one-way ANOVA. The primary outcome measure was the ICU mortality, which was compared in five groups of patients: no AKI, risk (R), injury (I), failure (F), and loss or end-stage (L/E).

## Results

Data from 722 patients were included, no AKI: 362 (50.1%), risk: 168 (22.9%), injury: 71 (9.8%), failure: 80 (11.1%) and loss or end-stage: 44 (6.1%). Patients were evenly matched with regards to age and sex. The ICU mortality was: no AKI (7.5%), risk (15.8%), injury (25.4%), failure (38.8%) and L/E (20.5%). The need for renal support also varied according to RIFLE criteria: no AKI (1.1%), risk (4.2%), injury (26.8%), failure (72.5%) and L/E (77.3%) (Table 1).

**Table 1:** Comparison among patients according to RIFLE criteria

Parameter of interest	No acute kidney injury (*n *= 362)	Risk class (*n *= 165)	Injury class (*n *= 71)	Failure class (*n *= 80)	Loss or end-stage renal disease (*n *= 44)
Mean age (years) ± SD	57.89 ± 18.2	59.68 ± 18.4	57.14 ± 16.7	56.51 ± 21.3	63.09 ± 14.7
Sex, males (%)	213 (58.8%)	96 (58.2%)	41 (57.7%)	41 (51.3%)	27 (61.4%)
Mean APACHE II score ± SD	10.68 ± 6.9	14.64 ± 7.6	19.7 ± 8.2	24.34 ± 8.1	16.91 ± 8
Mean PDR ± SD	16.09 ± 15.8	24.33 ± 19.2	37.13 ± 23.7	49.45 ± 23.4	30.66 ± 21
Mean SOFA score ± SD	3.39 ± 2.4	5.27 ± 3.3	7.15 ± 3.7	8.95 ± 3.4	5.39 ± 3.7
Need for inotropic support	56 (15.5%)	41 (24.8%)	28 (39.4%)	40 (50%)	14 (31.8%)
Need for renal support	4 (1.1%)	7 (4.2%)	19 (26.8%)	58 (72.5%)	34 (77.3%)
Need for mechanical ventilation	66 (18.2%)	43 (26.1%)	28 (39.4%)	32 (40%)	15 (34.1%)
Length of ICU stay, days	4.53 ± 5.2	5.25 ± 5.1	4.46 ± 4.8	6.44 ± 6.5	5.68 ± 6.2
Length of hospital stay, days	8.55 ± 7.1	9.2 ± 6.4	8.34 ± 7.1	10.76 ± 11.9	9.57 ± 7.1
ICU mortality	27 (7.5%)	26 (15.8%)	18 (25.4%)	31 (38.8%)	9 (20.5%)

## Conclusion

The RIFLE classification is a simple tool, which can be used to assess and classify AKI on admission to ICU. Moreover, it can reliably be used to predict and prognosticate outcome and need for renal support in ICU patients.
